# Identification of druggable hub genes and key pathways associated with cervical cancer by protein-protein interaction analysis: An in silico study

**DOI:** 10.18502/ijrm.v21i10.14536

**Published:** 2023-11-24

**Authors:** Azizeh Asadzadeh, Nafiseh Ghorbani, Katayoun Dastan

**Affiliations:** ^1^Department of Biology, Faculty of Science, Nour Danesh Institute of Higher Education, Meymeh, Isfahan, Iran.; ^2^Department of Microbiology, Faculty of Basic Sciences, Lahijan Branch, Islamic Azad University, Lahijan, Iran.

**Keywords:** Protein interactions, Cervical cancer, Hub genes, Gene expression, DEGs.

## Abstract

**Background:**

The uncontrolled growth of abnormal cells in the cervix leads to cervical cancer (CC), the fourth most common gynecologic cancer. So far, many studies have been conducted on CC; however, it is still necessary to discover the hub gene, key pathways, and the exact underlying mechanisms involved in developing this disease.

**Objective:**

This study aims to use gene expression patterns and protein-protein interaction (PPI) network analysis to identify key pathways and druggable hub genes in CC.

**Materials and Methods:**

In this in silico analysis, 2 microarray gene expression datasets; GSE63514 (104 cancer and 24 normal samples), and GSE9750 (42 cancer and 24 normal samples) were extracted from gene expression omnibus to identify common differentially expressed genes between them. Gene ontology and Kyoto encyclopedia of genes and genomes pathway analysis were performed via the Enrichr database. STRING 12.0 database and CytoHubba plugin in Cytoscape 3.9.1 software were implemented to create and analyze the PPI network. Finally, druggable hub genes were screened.

**Results:**

Based on the degree method, 10 key genes were known as the hub genes after the screening of PPI networks by the CytoHubba plugin. *NCAPG*, *KIF11*, *TTK*, *PBK*, *MELK*, *ASPM*, *TPX2*, *BUB1*, *TOP2A*, and *KIF2C* are the key genes, of which 5 genes (*KIF11*, *TTK*, *PBK*, *MELK*, and *TOP2A*) were druggable.

**Conclusion:**

This research provides a novel vision for designing therapeutic targets in patients with CC. However, these findings should be verified through additional experiments

## 1. Introduction

Cervical cancer (CC) is the fourth most common cancer among women worldwide (1). According to the database of research on cancer, CC affects more than half a million women and causes 0.3 million deaths annually (1, 2).

CC affects the lower part of the uterus, which is connected to the vagina. The progression of CC occurs slowly, taking up to 10 yr to become a precancerous lesion. On the other hand, the asymptomatic nature of CC makes patients unaware of it. Therefore, patients should perform a cervical screening test to discover the presence of cancer cells (1-3).

Symptoms of CC in advanced stages include vaginal bleeding, pelvic pain, prolonged menstrual bleeding, and pain during intercourse (3, 4). The main methods of treating CC are surgery and radiotherapy, however it may lead to posttreatment problems such as recurrence, metastasis, and drug resistance (2, 3).

Emphasized factors in the development of CC include co-infection with the pathogen, reproductive factors, increasing gene expression, or in some cases, decreasing gene expression in the cervix cell line, sexual behavior, obesity, smoking, and long-term use of hormones, and prevention of pregnancy (5).

The mechanisms involved in developing CC are very complex, and hub genes, RNAs, and different signaling pathways are related to it (6-8). Krüppel-like factor 4 and estrogen receptor 1 are closely related to the poor prognosis of patients with CC. Endothelin-3 and endothelin B receptors may play an important role in CC development (8). In a protein network, some nodes are defined as genes with high correlation in candidate modules, which are called hub genes. *CDC45, GINS2, MCM2, *and* PCNA*, have been reported as hub genes that are associated with the prognosis of CC patients (7).

One of the best biological networks that recently received much attention is protein-protein interaction (PPI) network analysis. This is an in silico method to identify proteins associated with various diseases. This method presents the useful information about how proteins interact with each other and their roles (9, 10).

Detection of differentially expressed genes (DEGs) in the first step of PPI analysis allows the identification of key biomarkers, which helps in the early diagnosis and treatment of CC and can potentially increase the patient's life expectancy. In addition, gene expression data helps identify pathways and molecular information to select effective targets for drug design (9, 11).

Our present study uses gene expression patterns and PPI network analysis to identify druggable hub genes and molecular pathways involved in CC.

## 2. Materials and Methods

### Selection of gene expression dataset 

This study was conducted by in silico analysis. Gene expression omnibus (GEO) is a widely used database for gene expression and RNA methylation profiling (12). In this database, searches were limited by study keyword, type of organism, study type, and entry type. In this step, 2 gene expression datasets for CC were obtained for further analysis.

### Data analysis and DEGs identification

DEGs between CC and normal samples in selected datasets were analyzed by GEO2R separately. Adjusted p-value < 0.05 as the cut-off criteria was determined. Up-regulated and down-regulated genes were selected based on log2 (FC) value > 1 and log2 (FC) value < -1, respectively. In the next step to exhibit the overlap of DEGs between the 2 datasets, a Venn diagram was drawn by Funrich software for up-regulated and down-regulated genes.

### Gene ontology (GO) and Kyoto encyclopedia of genes and genomes (KEGG) pathway analysis

GO assessment and KEGG pathway analysis were performed by EnrichR (13). For this purpose, all common up-regulated and down-regulated genes were used as input data in EnrichR.

### PPI network analysis

Online software STRING 12.0 was used to generate a PPI (14). To construct the network by STRING, interaction scores of < 0.4 were selected, and disconnected nodes were deleted. Finally, the network was formed by using text mining, neighborhood, experiments, gene fusion, databases, co-occurrence, and co-expression as active interaction sources. In the next step, for the PPI network visualization, Cytoscape software version 3.9.1 was used, and 10 key genes were detected by the CytoHubba plugin.

### Expression levels of hub genes in CC

To validate the differential expression levels of mRNA between CC and normal tissue, the online gene expression profiling interactive analyzer database was used.

### Candidate drugs for druggable hub genes 

Obtained hub genes were analyzed to search for their target drugs in the drug gene interaction database (https://www.dgidb.org/). Drug gene interaction databases from different sources, including ChEMBL, DrugBank, Ensembl, NCBI Entrez, and PharmGKB, were used to obtain candidate drugs (15, 16).

## 3. Results 

### Selection of gene expression dataset

Based on the following filtration: CC (study keyword), Homo sapiens (organism), expression profiling by an array (study type) and series (entry type), 2 datasets of GSE63514 and GSE9750 were obtained from NCBI-GEO. GSE63514 was based on GPL570 platforms (Affymetrix Human Genome U133 Plus 2.0 Array) and comprised 128 cervical specimens (104 cases and 24 controls), and GSE9750 was based on GPL96 platforms (Affymetrix Human Genome U133A Array) comprising 66 samples (42 cases and 24 controls).

### Data analysis and DEG identification

**Figure 1:**
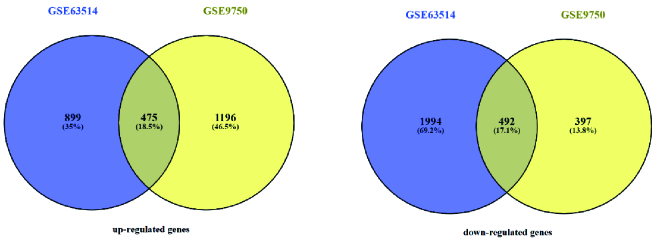
Venn diagram showed common DEGs between GSE63514 and GSE9750 datasets.

2 datasets of GSE63514 and GSE9750 were analyzed by GEO2R, separately. After ensuring the normal distribution of the data in the box plots, up- and down-regulated genes were identified. 1374 and 1671 genes in GSE63514, and GSE9750 were up-regulated, respectively. Among the studied genes, 2486 genes in GSE63514 and 889 genes in the GSE9750 dataset were down-regulated. Funrich software showed that 475 over-expressed genes and 492 down-regulated genes were common among GSE63514, and GSE9750 (Figure 1).

### GO and KEGG pathway analysis

The EnrichR database performed GO assessment and KEGG analysis. Table I shows the top 2 enriched GO biological processes and KEGG pathways. The common up-regulated DEGs were highly clustered in keratinocyte differentiation (GO: 0030216) and epidermal cell differentiation (GO: 0009913). The common down-regulated gene is involved in the DNA metabolic process (GO: 0006259) and DNA replication (GO: 0006260). KEGG 2021 human analyses revealed that common up-regulated DEGs were significantly enriched in the arachidonic acid metabolism and chemical carcinogenesis. KEGG pathways for down-regulated genes were cell cycle and DNA replication.

### PPI network analysis

**Figure 2:**
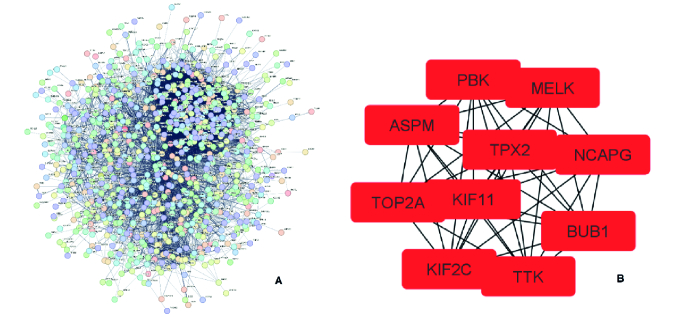
A) Overview of the protein-protein interaction network constructed by STRING. B) List of hub nodes obtained from PPI network by CytoHubba plugin.

Results from the PPI network analysis of the STRING showed 924 nodes and 13,688 edges (Figure 2A). Output DSV file of STRING visualized by Cytoscape 3.9.1 and analyzed. In PPI network with 924 nodes and 13,688 edges, 10 hub genes were determined by the CytoHubba plugin, which includes *NCAPG, KIF11, TTK, PBK, MELK, ASPM, TPX2, BUB1, TOP2A*, and *KIF2C *respectively, based on the rank (Figure 2B).

### Expression levels of hub genes in CC

**Figure 3:**
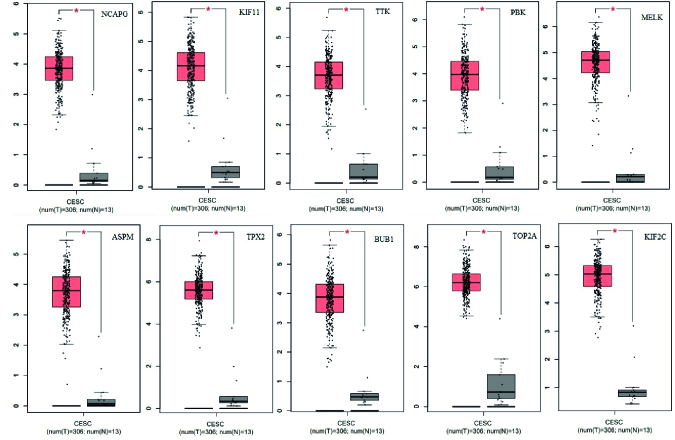
Box plots of differential expression levels of the hub genes, including *NCAPG, KIF11, TTK, PBK, MELK, ASPM, TPX2, BUB1, TOP2A*, and *KIF2C* in tumor (red) and normal tissues (gray).

To analyze the differential expression levels of the hub genes identified by the CytoHubba plugin, GEPIA was used. The mRNA expression levels of *NCAPG, KIF11, TTK, PBK, MELK, ASPM, TPX2, BUB1, TOP2A*, and *KIF2C *were increased in tumor tissue compared to those in normal tissues (num [T] = 306; num [N] = 13) (Figure 3).

### Candidate drugs for druggable hub genes 

The drug-gene interaction database is an online server that provides useful information about drug-gene interactions using publications, databases, and other sources (15, 16). The selected hub genes were subjected to the drug gene interaction database, and druggable hub genes were found. *KIF11, TTK, PBK, MELK,* and *TOP2A* were recognized as druggable genes among the studied genes. Candidate drugs are listed in table II.

**Table 1:** Top enriched GO biological processes and KEGG pathways


**Term**	**Adjusted p-value**	**Count**	**Genes**
**Common up-regulated DEGs (GO biological process)**			
**Keratinocyte differentiation** **(GO:0030216)**	3.72E-14	17	*FLG; SPRR3; ANXA1; WNT5A; KRT10; EVPL; EREG; TGM1; SCEL; SPRR2B; SPRR1A; TGM3; IVL; SPRR1B; EXPH5; EPHA2; S100A7*
**Epidermal cell differentiation** **(GO:0009913)**	3.05E-12	17	*FLG; SPRR3; ANXA1; SPINK5; WNT5A; KRT10; EVPL; EREG; TGM1; SCEL; SPRR2B; SPRR1A; TGM3; IVL; SPRR1B; EPHA2; S100A7*
**Common down-regulated DEGs (GO biological process)**			
**DNA metabolic process** **(GO:0006259)**	9.90E-44	67	*TOP2A; FEN1; MCM7; GMNN; NUDT1; HMGB2; HMGB3; MCM10; BRCA1; EXO1; CHEK1; NBN; TOPBP1; KPNA2; POLE; RFC5; WDHD1; HELLS; RFC3; RFC4; PARP1; LIG1; PARP2; TOP3A; SMC1A; MSH6; DBF4; TIMELESS; MCM3; MCM4; MCM5; DNA2; MCM6; MCM2; PRIM2; DNMT1; PCNA; RNASEH2A; PRKDC; PRIM1; TYMS; RECQL4; RAD51AP1; POLD3; ORC6; CDC45; CBS; RAD21; RAD54L; CDT1; MBD4; POLQ; TCF7L2; FANCL; FANCA; CDC7; CDC6; FANCG; RAD52; POLA1; RAD51; POLE2; CDK2; CDK1; RAD1; MNAT1; ATR*
**DNA replication** **(GO:0006260)**	1.77E-33	*FEN1; RNASEH2A; MCM7; MCM10; BRCA1*; *RECQL4*; *BRIP1; ORC6; CDC45; EXO1; CHEK1; NBN; CLSPN; TOPBP1; POLE; RFC5; WDHD1; TIPIN; RFC3; DUT; RFC4; RMI1; TOP3A; DONSON; CDC7; CDC6; POLA1; DBF4; POLE2; CDK2; TIMELESS; CDK1; MCM3; MCM4; MCM5; RAD1; DNA2; MCM6; ATR; MCM2*
**Common up-regulated DEGs (KEGG 2021 human)**			
**Arachidonic acid** **metabolism**	0.00323	9	*CYP2J2; CYP2C9; GPX3; EPHX2; PLA2G3; ALOX12; ALOX12B; ALOX15B; CBR3*
**Chemical carcinogenesis**	0.007507	*NOTCH2; EPHX2; UGT2B15; EPHX3; ARRB1; PIK3R1; CYP3A4; KLF4; CYP2C18; ESR1; CYP3A5; AR; CYP2C9; RPS6KA6; GSTA4; CCND1; PGR*
**Common down-regulated DEGs (KEGG 2021 human)**			
**Cell cycle**	7.65E-37	44	*GSK3B; PCNA; MCM7; PRKDC; BUB1B; TTK; PKMYT1; CDC20; CCNB2; CCNB1; ORC6; CDC45; PTTG1; RAD21; CHEK1; E2F1; E2F3; BUB3; SKP2; BUB1; CDKN2C; CDKN2A; CDC7; CDC6; CDC25C; SMC1A; CDC25A; CDC25B; CCNA2; DBF4; RBL1; CCNE2; TFDP2; CCNE1; CDK4; CDK2; CDK1; MCM3; MCM4; MCM5; MCM6; ATR; MCM2; MAD2L1*
**DNA replication**	2.85E-21	20	*RFC5; PRIM2; FEN1; RFC3; PCNA; RNASEH2A; RFC4; MCM7; LIG1; PRIM1; POLD3; POLA1; POLE2; MCM3; MCM4; MCM5; DNA2; MCM6; POLE; MCM2*
GO: Gene ontology, DEGs: Differentially expressed genes, KEGG: Kyoto encyclopedia of genes and genomes			

**Table 2:** Candidate drugs for *KIF11, TTK, PBK, MELK*, and *TOP2A* in treating CC


**Gene**	**Drug**
***KIF11***	Azd-4877, Litronesib, Filanesib, S-trityl-l-cysteine, Dimethylenastron, Ispinesib, Chembl392369, Chembl481931, Chembl409102, Sb-743921
***TTK***	Hesperadin, Bay-1217389, Bay-1161909
***PBK***	Chembl225519, Gefitinib, Linifanib, Chembl541400, Sp-600125
***MELK***	Cenisertib, Linifanib, Tae-684, Mln-8054, Pf-00562271, Dovitinib, Tozasertib, Sp-600125, Rg-1530, Gw843682x, Bms-387032, Chembl406845, Sb-220025, Sns-314, Ilorasertib, Ast-487, Jnj-7706621
***TOP2A***	Amrubicin, Epirubicin, Daunorubicin, C-1311, Mitoxantrone, Vosaroxin, Daunorubicin Citrate, Doxorubicin, Etoposide, Podofilox, Amsacrine, Berubicin Hydrochloride, Valrubicin, Etoposide Phosphate, Teniposide, Idarubicin, Idarubicin Hydrochloride, Mitoxantrone, Hydrochloride, Amrubicin Hydrochloride, Aldoxorubicin, Quercetin, Doxorubicin Hydrochloride, Daunorubicin Hydrochloride, Elliptecine, Chembl607534, Demethylzeylasterone, Chembl594695, Makaluvamine F, Adriamycin, Betulin, Chembl1773343, Chembl1080077, Carinatin G, Chembl594257, Chembl594153, Makaluvamine A, Ungeremine, Chembl2171812, Fisetin, Kaempferitrin, Chembl2332126, Frangulin B, Chembl594259, Diazirine, Elinafide, Chembl507986, Chembl2023733, Fluorouracil, 4'-o-acetylpatentiflorin B, Diphyllin, Makaluvamine C, Tfa Salt, Chembl244268, Makaluvamine E, 13-deoxydoxorubicin, Digitoxin, Chembl2171781, Vincristine, Amonafide, Chembl596082, Lupeol, Chembl2332127, Chembl594379, Chembl2332128, Huratoxin, Chembl593570, Idronoxil, Secaubryolide, Lycobetaine, Simocyclinone D8, Camptothecin, Genistein, Myricetin, Hydroquinone, Oleanderolide, Paclitaxel, Chembl2171794, Tricitrinol B, Dexrazoxane, Becatecarin, Gancotamab
*KIF11*: Kinesin family member 11, *TTk*: Tyrosine/threonine kinase, *PBK*: PDZ-binding kinase, *MELK*: Maternal embryonic leucine zipper kinase, *TOP2A*: Topoisomerase II alpha	

## 4. Discussion

CC is a serious problem in women's health. The slow progress of this type of cancer and the absence of symptoms in the early stages can cause delayed onset in diagnosis. In diseases related to the uncontrolled proliferation of cells, the molecular study of carcinogenesis mechanisms is important to achieve early detection methods and prevention of metastasis.

In silico approaches help us to achieve important results with low cost and time. In previous studies, we have shown that bioinformatics methods are a suitable option in the design of enzyme inhibitors, drugs, and vaccines (17-21).

Microarray technology and PPI networks, as in silico methods, are used to investigate cancer biomarkers and cellular mechanisms. Using these methods makes it possible to analyze gene clusters whose expression decreases or increases simultaneously. In this study, gene expression patterns and PPI network were studied to obtain key pathways and druggable hub genes in CC.

Common DEGs were analyzed by comparing the 2 datasets, GSE63514, and GSE9750 of CC obtained from the GEO database. 475 over-expressed genes and 492 down-regulated genes were identified. According to GO and KEGG pathway analysis, most DEG genes participated in keratinocyte differentiation, epidermal cell differentiation, DNA metabolic process, arachidonic acid metabolism, chemical carcinogenesis, cell cycle, and DNA replication pathways. The most important cause of CC is related to human papillomavirus infection, which infects epithelial cells, so the virus replication cycle is closely related to the differentiation process of infected keratinocytes (22). Targeting the arachidonic acid metabolism was used as a therapeutic method against CC (23). Among all DEGs in the PPI network, 10 hub genes were determined, which includes *NCAPG, KIF11, TTK, PBK, MELK, ASPM, TPX2, BUB1, TOP2A,* and *KIF2C*. Their differential expression levels were validated by the CytoHubba plugin and GEPIA, respectively. The role of all these genes has been discussed as cancer biomarkers. *NCAPG* is a prominent molecular target in many types of cancers, and overexpression of this gene plays an important role in carcinogenesis and tumor progression (24).

*KIF11* is a member of the kinesin family, and this gene has been over-expressed in tumor tissues. The functional study of *KIF11 *has shown that all stages of mitosis and cell division depend on it, and its main role is related to the formation and maintenance of bipolar spindle or cytokines (25). In a study by Zhou et al., on differentially expressed genes in adrenocortical carcinoma, they observed that compared to normal tissues, the expression of *KIF11* is significantly increased in adrenocortical carcinoma samples (26). Another gene found as a key gene in this study is tyrosine/threonine kinase (TTK), which is also effective in breast cancer, according to previous reports. Mishra et al., reported that hub genes, including *ASPM, BUB1, KIF2C, MELK, PBK*, and *TOP2A*, are oncogenes, and their expression is increased in all hepatocellular carcinoma samples (27). Tpx2, in addition to regulating the mitotic spindle, plays an important role in cell-cycle kinase Aurora A activation, and its overexpression is associated with the development of various cancers (28). For this reason, in many studies, TPX2 has been proposed as a marker for the diagnosis and prognosis of malignancies (29). This research confirms the validity of the key genes obtained in this study.

Finally, among the studied genes, *KIF11, TTK, PBK, MELK, *and *TOP2A* were recognized as druggable genes. Most obtained drugs are inhibitors, and in many researches, their anti-cancer roles have been mentioned (30). Litronesib exerts its antitumor activity by selectively inhibiting the mitosis-specific kinesin Eg5 (31). Hesperadin is an aurora kinase inhibitor. Amrobicin and idronoxil are 2 potent inhibitors of topoisomerase II (32).

The most important drugs approved for the treatment of CC are bevacizumab, topotecan hydrochloride, pembrolizumab, and bleomycin sulfate. Bevacizumab is a class of monoclonal antibodies that work by binding to the VEGF protein, and it blocks the growth of blood vessels around the tumor. The antitumor mechanism of topotecan hydrochloride and bleomycin sulfate is related to binding to the DNA of cancer cells and other rapidly growing cells. Pembrolizumab binds to the PD-1 protein on the surface of T cells to attack and kill cancer cells (33-36).

## 5. Conclusion

This study identified 967 genes (475 over-expressed and 492 down-regulated genes) as DEGs in CC. Our results showed that key genes including *NCAPG, KIF11, TTK, PBK, MELK, ASPM, TPX2, BUB1, TOP2A,* and *KIF2C* might have effective roles in CC. In addition, drug compounds targeting the hub gene were identified. These drugs can potentially be used to treat patients with CC. However, future validation by both in vitro and in vivo studies is inevitable.

##  Conflict of Interest

The authors declared that there is no conflict of interest.
